# A Pelvic Hernia Through Two Defects in the Pouch of Douglas in a Patient With Peritoneal Xanogranuloma: A Report of a Very Rare Case

**DOI:** 10.7759/cureus.56808

**Published:** 2024-03-24

**Authors:** Nimah A Rabai, Najih Khaldi, Arqam Alrababah, Wissam A Marji, Mu'ayyad M Mugdadi

**Affiliations:** 1 Department of General Surgery, Princess Basma Teaching Hospital, Irbid, JOR; 2 Department of General Surgery, Abdali Hospital, Amman, JOR; 3 Department of Radiology, Princess Basma Teaching Hospital, Irbid, JOR; 4 Department of General Surgery, Jordanian Royal Medical Services, Irbid, JOR

**Keywords:** small bowel obstruction, peritoneal xanthogranuloma, rectouterine pouch, pouch of douglas, pelvic hernia, internal hernia

## Abstract

Pelvic internal hernias, including pouch of Douglas hernias, are a very rare cause of small bowel obstruction. They pose a challenge in diagnosis due to their rarity and lack of specific radiological features. The definitive diagnosis is usually reached intraoperatively. The treatment consists of reduction with or without resection of the herniated bowel and primary repair of the defect. Mesh placement has been reported but is still arguable, as no musculofacial defect is involved. Here, we present a case of a 28-year-old female patient, a nulliparous with multiple medical conditions including familial Mediterranean fever (FMF) and an extremely rare tumor, peritoneal xanthogranuloma. She had a history of laparoscopic left ovarian cystectomy, and complained of abdominal pain and distention for three days prior to admission. Her symptoms were associated with constipation and recurrent vomiting and she was admitted as a suspected case of small bowel obstruction. CT scan suggested the possible diagnosis of a pelvic hernia, yet the definitive diagnosis was reached intraoperatively after noticing the presence of two defects on the left side of the pouch of Douglas. A primary repair of the defects was performed after reduction of the viable herniated bowel. The patient was discharged on the third postoperative day with uneventful course of recovery.

## Introduction

Internal hernias are an uncommon cause of small bowel obstruction (SBO), with an incidence of 0.6-5.8% of SBO cases. Many types of internal hernias have been identified and were classified according to the anatomical location of the defect [1].

The preoperative diagnosis is usually challenging, due to the rarity of the condition and the lack of characteristic radiological features [2]. With a high index of suspicion, surgeons can provide the proper management in a timely manner and avoid the dire complication of bowel ischemia. Here, we present a case of a young female patient with a diagnosis of an extremely rare peritoneal tumor, peritoneal xanthogranuloma, and a history of gynecological laparoscopic surgery, who presented with symptoms of SBO. The definitive diagnosis of pouch of Douglas (POD) hernia was reached intraoperatively, and managed with reduction and primary repair. We hope our case will add knowledge to previously published literature regarding the two very rare conditions, POD hernia and peritoneal xanthogranuloma.

## Case presentation

A 28-year-old single nulliparous female patient, a known case of hypertension (HTN), familial Mediterranean fever (FMF), idiopathic tachycardia, and osteopenia, presented to the emergency department (ED) complaining of diffuse abdominal pain for three days. The pain started in the epigastric area, then became diffuse with abdominal distention and recurrent vomiting. Her symptoms were also associated with constipation; however, the patient passed stool a few hours before presenting to the ED.

Her past surgical history included open splenectomy for symptomatic splenomegaly in 2014, and laparoscopic left ovarian cystectomy in 2016, which included an excisional biopsy of an incidental peritoneal mass which was later diagnosed as peritoneal xanthogranuloma. The patient was on a regular beta-blocker (propranolol 20 mg once daily), calcium carbonate tablet 500 mg twice daily, alfacalcidol capsule once daily, and colchicine tablet 1 mg twice daily. She had no known allergies.

On physical examination, Her vital signs were within normal ranges except for mild tachycardia; the abdomen was distended and tympanic with tenderness all over. There was a left subcostal scar consistent with her history of open splenectomy, and multiple trocar scars related to the laparoscopic left ovarian cystectomy. No abdominal wall hernias, incisional or inguinal/femoral hernias were palpable. On digital rectal examination, there was a small amount of soft stool present in the rectum. A blood workup was requested for the patient, in addition to an abdominopelvic CT scan.

The patient was admitted to the surgical ward as a suspected case of SBO. She was kept Nil Per Os (NPO) on IV (Intravenous) fluids. A nasogastric tube (NGT) was inserted to decompress the stomach, and she was started on IV antibiotics and analgesics awaiting the blood workup and CT scan results. Her lab tests were significant for elevated white blood count (WBC) 17.02 x 10^3^/uL and thrombocytosis (platelets count 683 x 10^3^/uL) which could be explained by her splenectomised status. She had normal hemoglobin level, kidney function test, and electrolyte levels (Table 1). 

**Table 1:** Laboratory workup on admission. WBC, white blood cells; HgB, hemoglobin; MCV, mean corpuscular volume; PLT, platelets

Parameter	Value	Reference Range
WBC	17.02 x 10^3^ /µL	4.0-11.0 x 10^3^ /µL
HgB	12.7 g/dL	13.8-17.2 g/dL for males, 12.1-15.1 g/dL for females
MCV	77.6 fL	80-96 fL
PLT	683 x 10^3^ /µL	150-400 x 10^3^ /µL
Glucose	6.21 mmol/L	3.9-6.1 mmol/L
Urea	3.2 mmol/L	2.5-6.7 mmol/L
Creatinine	41 µmol/L	59-104 µmol/L for males, 45-90 µmol/L for females
Sodium (Na+)	138 mmol/L	136-145 mmol/L
Potassium (K+)	4.67 mmol/L	3.5-5.1 mmol/L

**Figure 1:**
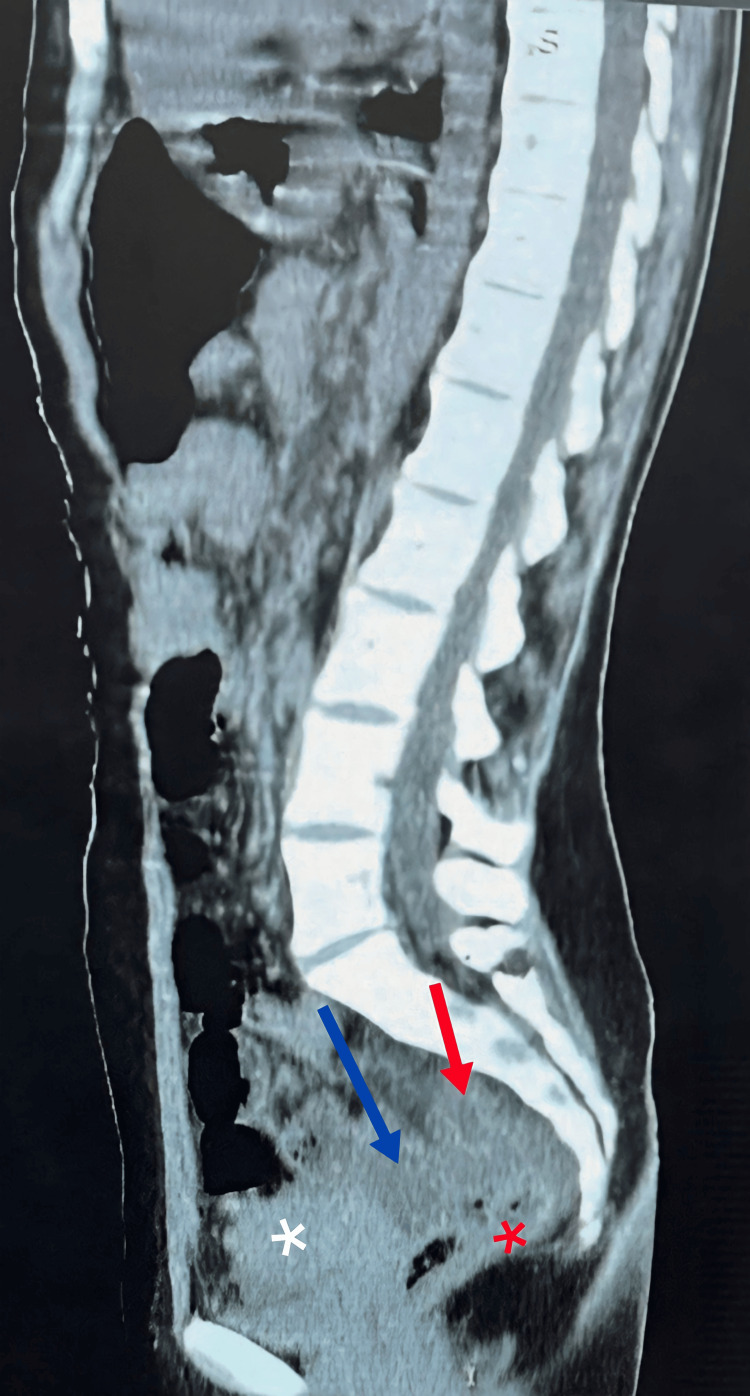
Abdominopelvic CT scan (sagittal view) shows the suspected herniated bowel segment (red arrow) between the uterus and rectum in addition to the presence of pelvic free fluid (blue arrow). Uterus (white asterisk), rectum (red asterisk)

The abdominopelvic CT scan report stated the presence of a fluid-density structure in the pelvis that could be continuous with the small bowel, or could represent a focal fluid collection (Figure 1). For confirmation the radiologist recommended repeating the scan with oral and IV contrast.

**Figure 2:**
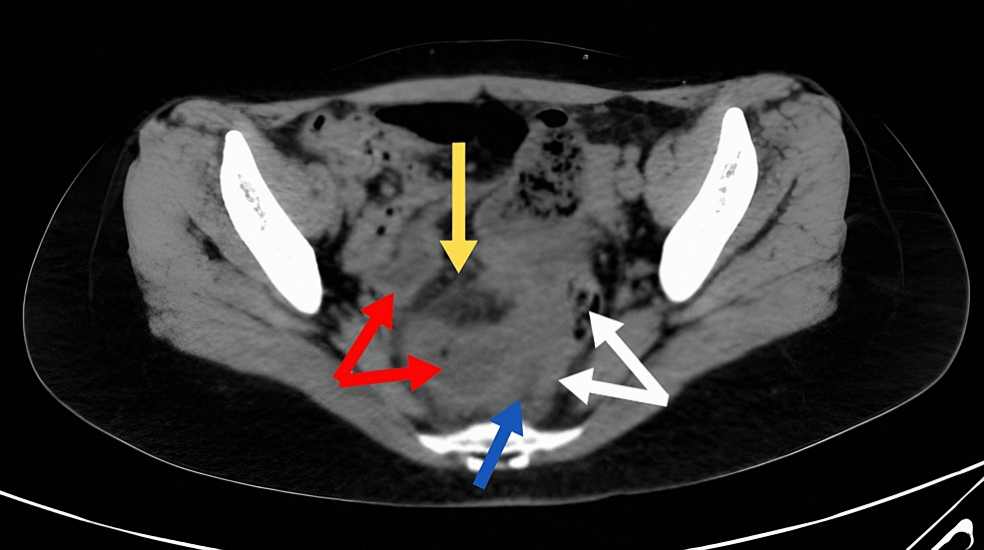
Abdominopelvic CT scan (axial view) shows the presence of dilated small bowel loops with thick wall (red arrows) in proximity to the sigmoid colon (white arrows) surrounded by pelvic free fluid (blue arrow) and dirty mesenteric fat (yellow arrow).

There were multifocal areas of wall thickening in both the small and large bowel in association with vascular engorgement and multiple enlarged mesenteric lymph nodes which were suspicious of Crohn’s disease. Small amount of free fluid was also present in the pelvis (Figure 2).

Taking into consideration the previous surgical history of the patient, the mild improvement after the insertion of the NGT and the initiation of IV fluids and antibiotics, in addition to the radiological suspicion of a complicated case of Crohn’s disease, despite the lack of a previous diagnosis, our final preoperative impression was of a partial intestinal obstruction that could be related to adhesions from the previous surgeries or due to complications of undiagnosed Crohn’s disease. So, we initially treated the patient conservatively with serial evaluation.

On the second day of admission, the patient was still experiencing vomiting, abdominal pain, and distention; in addition, her WBC was still elevated. This was considered a failure of the conservative management and a decision was made with the patient's consent to perform exploratory laparotomy. 

**Figure 3:**
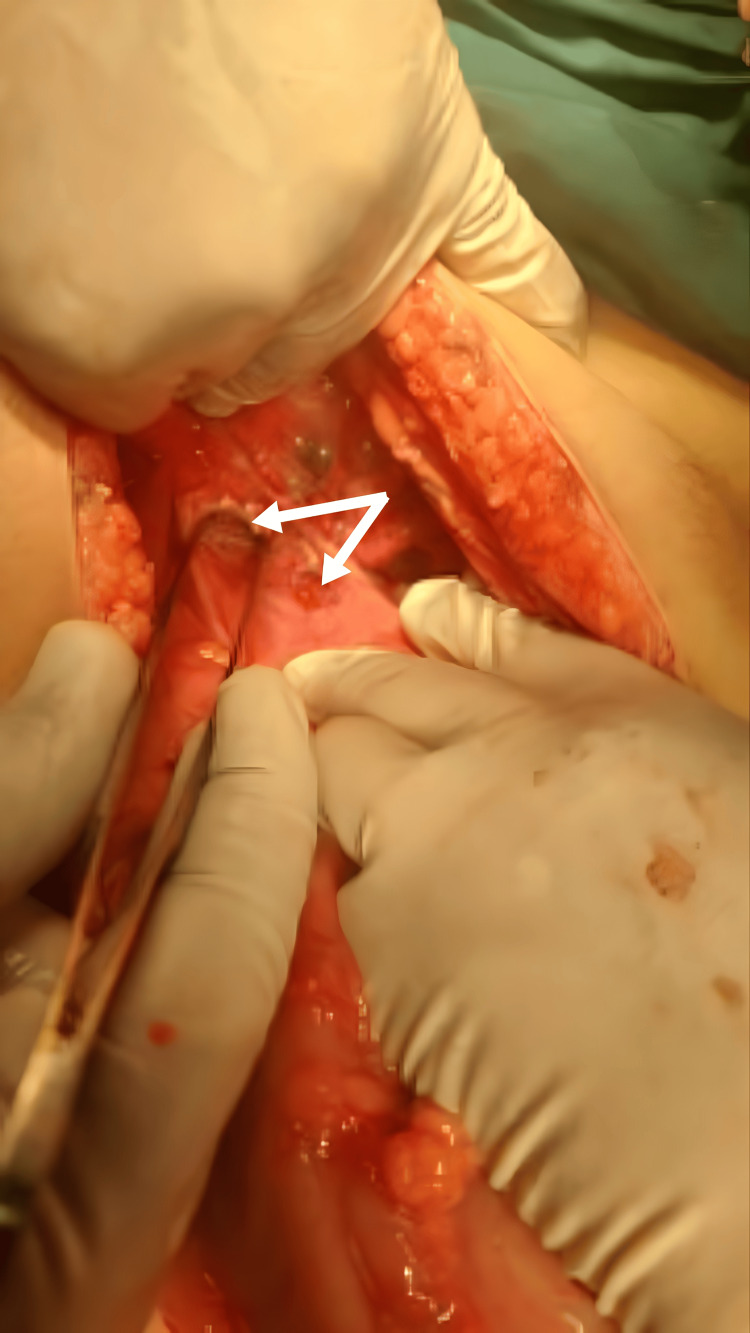
Two defects seen on the left side of the pouch of Douglas.

Upon entering the abdominal cavity, the small bowel was dilated down to the distal ileum, with a segment of distal ileum protruding through two defects in the left side of POD, the rest of the distal ileum and colon were collapsed. The defects in POD measured approximately 2 cm and 1.5 cm (Figure 3).

**Figure 4:**
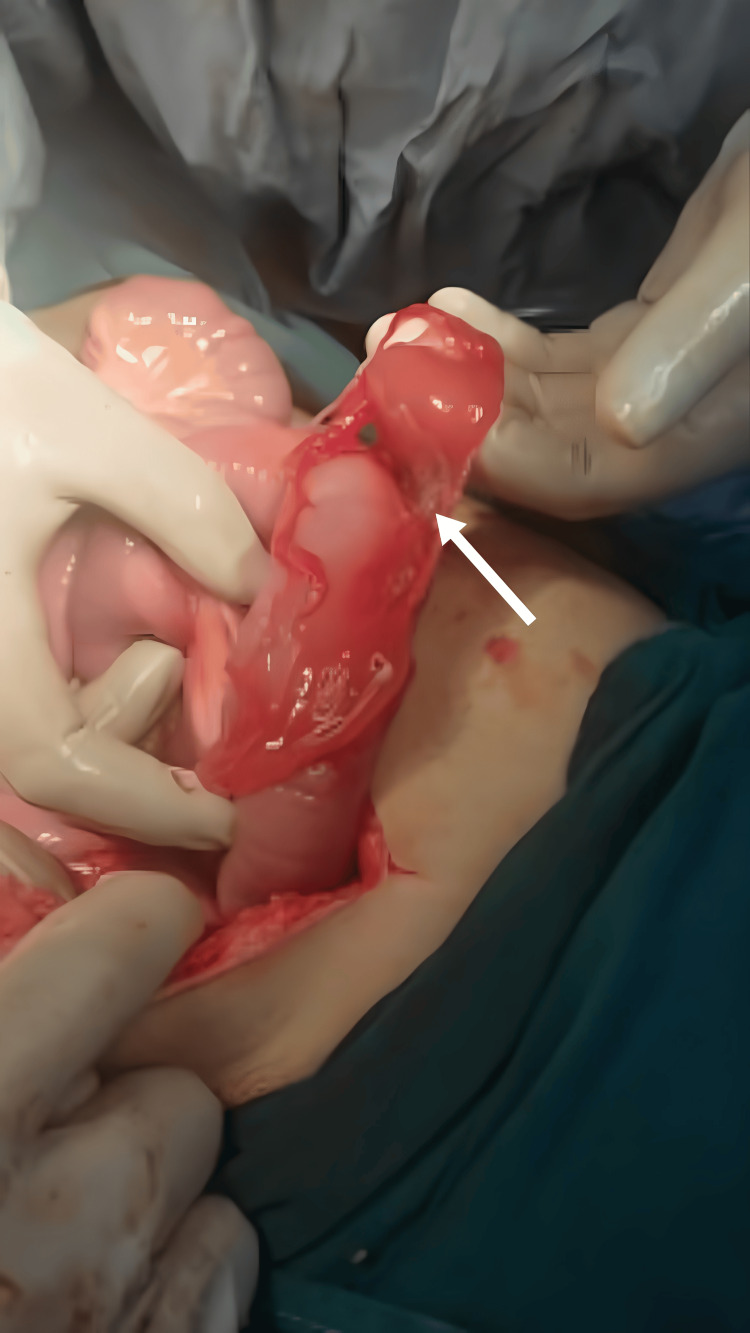
Gel-like material adherent to small bowel loops.

There were deposits of gel-like material on different segments of the small bowel (Figure 4), with surrounding adhesions.

**Figure 5:**
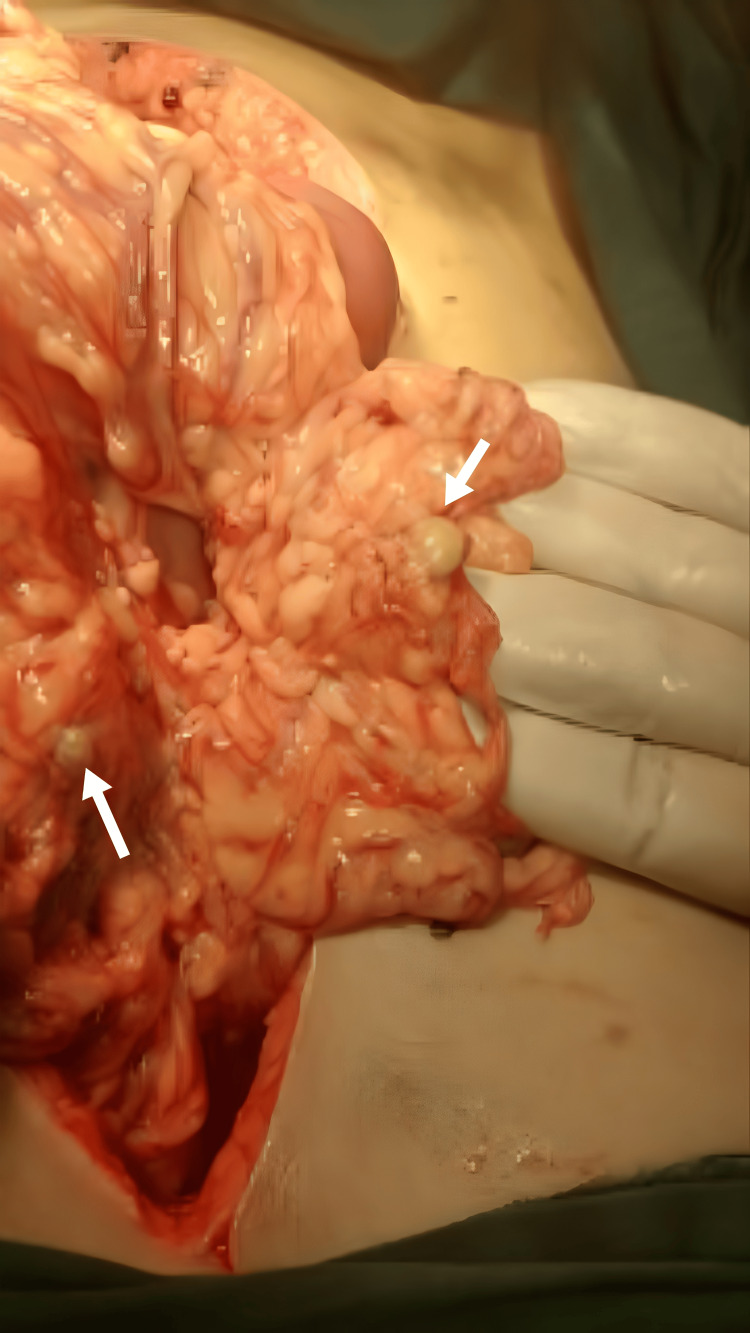
Multiple small yellow nodules seen on the omentum.

Multiple small round yellow lesions were seen scattered all over the omentum (Figure 5) with multiple adjacent omental defects present.

Partial omentectomy including the defects and samples of the lesions was performed and sent for histopathological examination. Multiple cystic lesions with yellow gel-like material were seen on the uterus and the left ovary. These lesions, as well as, the omental lesions, could be related to the patient’s previous diagnosis of peritoneal xanthogranuloma; still, biopsies were taken for histopathological confirmation. We also noted the presence of enlarged mesenteric lymph nodes and we sampled a few nodes for histopathological examination.

The herniated segment of the bowel looked healthy with mild erythema but no signs of ischemia, so reduction of the hernia was performed with no need for resection, and the POD defects were repaired with simple primary closure. Although the appendix looked grossly normal, appendectomy was performed to rule out a missed cause for the multiple gel-like (mucinous) lesions seen in the abdomen, namely, mucinous neoplasms. A drain was placed in the pelvis followed by closure of the abdominal wall layers.

On histopathological examination, the omental, uterine, and left ovarian lesions were consistent with the patient’s previous diagnosis of xanthogranuloma and contained mesothelial-lined tissue infiltrated with sheets of foamy histiocytes, with no evidence of dysplasia or malignancy, and the fragments of ovarian tissue were unremarkable. The appendix had no morphological signs of acute appendicitis or para-appendicitis and was negative for dysplasia or malignancy. The sampled lymph nodes were reactive with no evidence of malignancy.

The patient had an uneventful postoperative period. On the second postoperative day, she passed flatus and was started on oral fluids. The pelvic drain contained around 25 cc of serous fluid and was removed. She was discharged home on the third postoperative day and had an uneventful course of follow-up at the outpatient clinic.

## Discussion

Internal hernias are a rare cause of SBO, contributing to only 0.6-5.8% of the cases, but with a mortality rate reaching 50% secondary to strangulation and ischemia [3,4]. They are classified according to the anatomical location where the defect occurs [5]. The paraduodenal type is the most common type, contributing to 50% of reported internal hernias [5,6].

One of the rare types of internal hernias is pelvic hernia, accounting for only 7% of internal hernias [1]. It includes hernias through the broad ligament, the perirectal fossa, and hernias through POD [5]. Historically, pelvic hernias were defined as hernias occurring due to weakness in pelvic floor muscles; however, this definition does not completely apply to pelvic hernia due to a defect in POD with no associated pelvic floor weakness [1,2], as in the case we present here.

The pouch of Douglas, also known as the rectouterine pouch, is part of the peritoneal coverage in the pelvis that reflects between the uterus and the rectum [5]. The incidence of a defect with subsequent hernia in this site is very rare and can be related to both congenital and acquired causes. Some of the predisposing factors for acquired pelvic internal hernias include old age, pelvic surgeries, trauma, and inflammatory process, in addition to pregnancy and delivery [2,4,5,7]. Our patient was young, nulliparous, and had two inflammatory conditions, FMF and peritoneal xanthogranuloma, and a previous gynecological laparoscopic surgery for the removal of a left ovarian cyst.

Upon reviewing the published literature on POD hernia on Pubmed, a few reported cases were found describing this unique entity of pelvic hernias. The age range of the reported cases was 17-80 years, a surgical history of hysterectomy was appreciated as the cause of hernia in the elderly patients, while a congenital etiology for the defect was the cause in the young patients with no previous surgeries [2,3] Our patient was young but had a previous laparoscopic left ovarian cystectomy, which explains the site of the defects seen intra-operatively.

The diagnosis of POD hernia is usually reached intraoperatively; however, imaging can aid in reaching a preoperative diagnosis. A CT scan clue that could be suggestive of the diagnosis is the presence of bowel loops behind the uterus in the pelvic region in a background of bowel obstruction including findings of dilated bowel loops with air-fluid levels. Still, it’s hard to differentiate POD hernia from other types of pelvic hernias based on this sole finding [2,5,8]. In our case, a preoperative diagnosis of a pelvic hernia was suggested by the radiologist after suspecting the presence of bowel loops in the pelvis between the uterus and the rectum, despite having only a CT scan without contrast due to lack of contrast medium at that particular time. However, other findings seen on the scan related to the peritoneal xanthogranuloma, mainly the thickening of the bowel wall and increased vascularity, made the radiologist suspicious of undiagnosed inflammatory bowel disease (IBD) as the culprit in this case.

The rarity of POD hernias and the lack of specific imaging characteristics are the main challenges in reaching a preoperative diagnosis. Thus, a high index of suspicion is required by the surgeons to provide the proper diagnosis and management and to avoid the ominous progression into bowel ischemia [2,5,8].

In treating POD hernias, both open and laparoscopic techniques were successfully described in the published cases. Reduction and primary closure of the defect were usually performed. The use of mesh was described in a case by Bunni et al. [6]. Nevertheless, its use is arguable, as no musculofacial defect is involved [1,2,7]. In two published cases, herniotomy and marsupialization were performed instead [3,9]. In our case, we performed reduction with primary closure. No resection was needed as the herniated segment of the bowel seemed viable with no signs of ischemia. Subsequently, our patient had an uneventful course of follow-up after surgery.

Finding two defects in the POD in this patient, in addition to her previous diagnosis of peritoneal xanthogranuloma, which is an extremely rare tumor, makes this case very rare and unique. Only one case report was found on Pubmed search for peritoneal xanthogranuloma published in English [10]. The hallmark of this tumor is the presence of a mixture of foamy cells with both acute and chronic inflammatory cells within the multiple yellow nodules that form in the abdomen [10]. Based on its inflammatory pathogenesis, it could theoretically have a role in the defect formation in this case, yet it’s an extremely rare tumor and its potential risk of causing perforation or hernia defect is still unknown. Future research may elucidate the possible short- and long-term complications of this tumor.

## Conclusions

SBO is a common surgical diagnosis, yet a high index of suspicion is needed to diagnose the very rare cases caused by pelvic internal hernias, including the pouch of Douglas hernia. Physicians should be knowledgeable of this etiology to provide a timely diagnosis and proper treatment of suspected cases, as delay in management leads to the development of bowel ischemia and is associated with a high mortality rate. Although inflammatory causes have been associated with pelvic hernia, the role of peritoneal xanthogranuloma in hernia defect formation is unclear as yet. Future studies of this extremely rare tumor might help us further understand its pathogenesis and possible complications.
